# A semantic web framework to integrate cancer omics data with biological knowledge

**DOI:** 10.1186/1471-2105-13-S1-S10

**Published:** 2012-01-25

**Authors:** Matthew E Holford, Jamie P McCusker, Kei-Hoi Cheung, Michael Krauthammer

**Affiliations:** 1 Interdepartmental Program in Computational Biology & Bioinformatics, Yale University, 300 George Street, Suite 501, New Haven, CT, 06511, USA; 2 Department of Pathology, Yale University, 310 Cedar Street LH 108, PO Box 208023, New Haven, CT, 06520, USA; 3 Department of Computer Science, Yale University, PO Box 208285, New Haven, CT, 06520-8285, USA; 4 Center for Medical Informatics, Yale University, 300 George Street, Suite 501, New Haven, CT, 06511, USA; 5 Department of Genetics, Yale University, 333 Cedar Street, New Haven, CT, 06520, USA

## Abstract

**Background:**

The RDF triple provides a simple linguistic means of describing limitless types of information. Triples can be flexibly combined into a unified data source we call a semantic model. Semantic models open new possibilities for the integration of variegated biological data. We use Semantic Web technology to explicate high throughput clinical data in the context of fundamental biological knowledge. We have extended Corvus, a data warehouse which provides a uniform interface to various forms of Omics data, by providing a SPARQL endpoint. With the querying and reasoning tools made possible by the Semantic Web, we were able to explore quantitative semantic models retrieved from Corvus in the light of systematic biological knowledge.

**Results:**

For this paper, we merged semantic models containing genomic, transcriptomic and epigenomic data from melanoma samples with two semantic models of functional data - one containing Gene Ontology (GO) data, the other, regulatory networks constructed from transcription factor binding information. These two semantic models were created in an ad hoc manner but support a common interface for integration with the quantitative semantic models. Such combined semantic models allow us to pose significant translational medicine questions. Here, we study the interplay between a cell's molecular state and its response to anti-cancer therapy by exploring the resistance of cancer cells to Decitabine, a demethylating agent.

**Conclusions:**

We were able to generate a testable hypothesis to explain how Decitabine fights cancer - namely, that it targets apoptosis-related gene promoters predominantly in Decitabine-sensitive cell lines, thus conveying its cytotoxic effect by activating the apoptosis pathway. Our research provides a framework whereby similar hypotheses can be developed easily.

## Background

The Yale Specialized Program in Research Excellence (SPORE) in skin cancer is a large translational cancer project, which aims to accelerate the movement of biological insights from the "bench to bedside". The SPORE collects skin cancer samples from mostly malignant melanoma patients and performs a multitude of Omics studies, probing the melanoma genome, epigenome, transcriptome and proteome. This data can be integrated with clinical outcome information to derive prognostic and predictive biomarkers, i.e. genomic markers that predict patient survival and drug therapy effectiveness, respectively.

Conventionally, these markers are either derived statistically in an unbiased fashion [1], or by prior knowledge and candidate (gene) selection [2]. We are interested in combining these approaches, and are developing means for unbiased assessment of Omics data using existing knowledge on cellular processes that affect drug effectiveness. The representational inclusivity of semantic models simplifies such heterogeneous methodologies. Here we create semantic models that define the genomic state of cancer cells and the functional annotation of the cells' molecular entities (i.e. genes or proteins). We query these semantic models using SPARQL to better understand the molecular basis of drug resistance and sensitivity.

We start by retrieving quantitative data from a large relational database, a component of the Corvus architecture [3], storing melanoma Omics data. To do this, we created a new semantic component of Corvus, a SPARQL endpoint which relies upon Hibernate [4] for Object Relational Mapping (ORM). Through this endpoint, we can dynamically create semantic models of the data stored within. We can then merge these quantitative semantic models with other semantic models holding systematic biological information. The Omics data is thus annotated with functional information, such as involvement in certain cellular processes, hierarchical classification or membership in a set of similarly delineated biological entities. Currently, these semantic models of functional data are (1) SKOS-converted GO [5] information and (2) representations of transcription factor binding networks. Though these semantic models are necessarily ad hoc, they were created to support a common interface, namely the pointing of annotative information to a gene or protein specified by a universally recognized identifier.

As a case study, we used the new Corvus SPARQL endpoint to create a semantic model of data representing drug response to Decitabine, a demethylating agent that has been shown to be clinically active in melanoma [6]. Using SPARQL, we queried Corvus for melanoma samples with information on promoter methylation status and gene expression before and after Decitabine treatment. This semantic model is augmented with functional annotations using the GO and transcription factor binding network semantic models. The resulting combined semantic model is then queried to find molecular mechanisms that explain why some samples have better response to Decitabine treatment than others.

To attain these goals, we needed to build a data structure that integrated quantitative Omics data with functional information. Our combined data structure incorporates gene expression and methylation data for seven melanoma cell lines [7]; it also contains GO annotations for the whole of the human genome and networks of genes within the sphere of influence of known human transcription factors. Expressing this data structure as a semantic model affords us a number of advantages. First, it provides a way for others to borrow from and build upon our work. It allows us to use the standardized SPARQL interface to perform queries that bridge quantitative and functional knowledge. It also gives us the capability to infer previously unstated information by reasoning over the data with a Semantic Web aware Description Logic (DL) reasoner. We attempted wherever possible to borrow terms from well-established OBO ontologies [8].

Doing so places our work under the auspices of community defined best practice and allows our model to be used in conjunction with similarly designed semantic models. Building the model involved the use of a variety of cutting-edge Semantic Web technologies and required the creation of several novel tools. The work proceeded along three lines: (i). Creation of quantitative semantic models by conversion of relational data from melanoma cell lines to RDF/OWL; (ii). Creation of ad hoc semantic models of functional data to represent information from GO and transcription factor networks; and (iii). Integration of the two through the common interface provided by the semantic models of functional data.

The integration of quantitative and functional biological information to infer relevant new information has been frequently explored. BioBIKE offers an environment for users to integrate a wide variety of experimental and genomic data to reach new conclusions [9]. Originally released as a LISP interactive library [10], the software is now web-based to accommodate users without programming expertise. When combined with the BioDeducta module, it enables automated reasoning [11]. Although BioBIKE makes extensive use of ontologies, it is not currently Semantic Web enabled. Another notable example is HyBrow, a tool for the generation and evaluation of biological hypotheses [12]. Here, the user can derive hypotheses from HyBrow's knowledge base of functional biological information and test them using various high-throughput data sources. The latest incarnation, HyQue [13], uses OWL to create a Semantic Web representation of its underlying knowledge base. Like the work presented here, it allows the testing of hypotheses against a genomic knowledge base through SPARQL queries. It does not, however, provide a direct means of incorporating new quantitative experimental data with its knowledge base.

Chem2Bio2RDF [14] offers a similar integration of chemical knowledge repositories using Semantic Web technology. It also offers a SPARQL endpoint, but lacks the means to incorporate raw experimental data. The opposite is true of a recent endeavor by Song et al [15]. Here, OWL is used to provide an integrated model of quantitative microarray data. Like us, they use a dynamic mapping model (in this case, d2rq [16]) to provide semantic access to relational data. Their project currently does not incorporate additional functional genomic knowledge, however. Additionally, it currently only supports tissue microarry (TMA) data, whereas Corvus can potentially store any kind of high-throughput quantitative biological data. Recent efforts by the National Cancer Institute as part of the caBIG initiative [17] have focused on addressing the integration issue through the use of an Extraction-Transform-Load (ETL) strategy. Notably, the caIntegrator2 [18] project uses ETL to integrate quantitative Omics data from caArray [19] and functional biological data from caBio [20]. The Bio2RDF project is notable for providing normalized Uniform Resource Identifiers (URI)s for a wealth of identifiers and relationships from functional biology in the hopes of allowing easier integration of diverse data sets [21].

Our previous work is discussed in [22]. This paper offers several significant advances. The functional knowledge component of our semantic model is enhanced by inclusion of information about transcription factor networks, proving the extensibility of our interface with existing biological data sources. Data about transcription factor networks is not typically included in integrated genomics models. Their inclusion was facilitated by the transitive reasoning capaiblities of the Semantic Web technology. We show several new example applications of our integrated semantic model. We examine genes potentially targeted by Decitabine in light of the biological pathways with which those genes are associated, comparing apoptotic genes with those genes involved with a divergent process - cell proliferation. Additionally, we try to find Decitabine-targeted genes that express particular transcription factors. In all cases, we use statistical tests to evaluate the significance of our results.

## Results and discussion

### The Corvus SPARQL endpoint

#### Rationale for building a SPARQL endpoint

Data for seven melanoma cell lines was stored in a relational database component of Corvus, an object model for experimental data. It currently controls a data warehouse holding over 4 million observations from diverse Omics experiments across melanoma cell lines. Presently, Corvus exists as a Java library with object-relational mapping (ORM) accomplished through Hibernate. Quantitative cancer omics data is stored in a standard database schema specified by the ORM. We present here a new semantic interface to Corvus which retrieves data in the form of RDF triples. Unfortunately, the sheer volume of data contained within our local Corvus database would result in a semantic model of such size as to be untenable for the purposes of DL reasoning. What was needed instead was a way to retrieve a subset of the Corvus warehouse containing only the information essential to the problem at hand. Ideally this could be accomplished in a dynamic fashion.

Integration of traditional relational databases with RDF has been explored extensively in recent years [23]. Typically the approach is to create a generic mapping between relational and RDF schema. This has been done either through automatic mappings, where relational tables correspond to RDFS classes and relational columns to RDF predicates [24], or with domain-specific semantics [25]. Some tools, such as d2rq, provide for both approaches and allow user customization for complex cases such as when mappings are not one-to-one. Mappings may be stored in a variety of formats, ranging from XML configuration files to custom languages such as R2O [26]. These mapping artifacts can then be used to dynamically generate SQL queries to the relational database based upon queries expressed according to the RDF schema, usually using SPARQL.

We experimented directly with the d2rq framework, which allows a relational database to be queried like a semantic model using SPARQL. Using a configuration file to map fields in the Corvus object model to RDF properties, we were able to generate SPARQL queries that retrieved a manageable subset of the Corvus warehouse. However, we found that the SQL generated by the tool to query the relational database was inefficient and data retrieval took longer than expected. We decided instead to leverage the Hibernate mappings already part of the Corvus object model to interact with the relational database. We wrote a SPARQL interface to the Corvus object model which interacts directly with the Java/ORM library, taking advantage of Hibernate's ability to optimize and cache relational queries. To the best of our knowledge, although the issue of mapping SPARQL to object oriented representations such as Hibernate has been discussed [27,28], no tools for doing this have been released to the public. Our approach is to create wrapper classes around the Hibernate mapping classes which map the property getters to RDF predicates. Indirect mappings make possible situations in which the RDF and relational schemas do not correspond one to one. Though this approach is not necessarily a universal solution, we felt that given Corvus' ability to represent such a broad swathe of Omics data, the performance gain offered by these customized mappings more than justified the up-front expense of their creation.

#### Corvus model to RDF mapping

**Figure 1:**
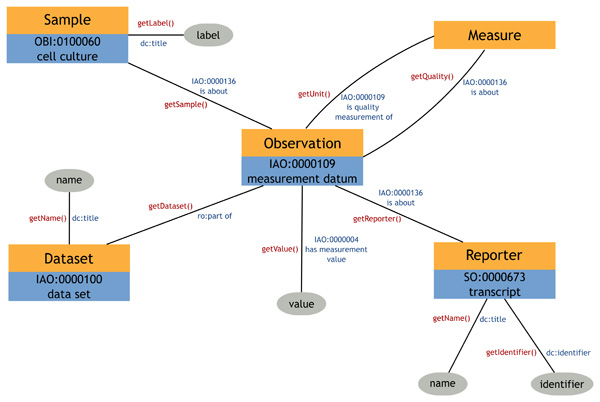
**Corvus object model**. Diagram showing Java classes in the Corvus model (orange boxes) next to their corresponding OWL classes (blue boxes). Data or annotation properties are shown as gray ellipses. Edge labels show the Java method used to call the Corvus model in red and the RDF property used in the semantic model in blue.

We mapped fields from the Corvus object model to classes and relationships from OBO ontologies. In particular, we employed terms from Information Artifact Ontology (IAO) [29] and Ontology for Biomedical Investigations (OBI) [30]. In addition to being actively developed, these ontologies are notable for building upon the foundation Basic Formal Ontology (BFO) [31] and the OBO Relation Ontology (RO) [32] which were specially designed to be extensible by any biomedical ontology. This allows our semantic model to be incorporated with other OBO ontologies with relative ease. It should be noted that we are simply borrowing terms from these ontologies, not incorporating them in their entirety as doing so would have a significantly deleterious effect on reasoning performance. This does not pose a hindrance to our goals as we do not need to make inferences across the whole hierarchy of terms in these ontologies. By using the terms, however, we provide an entry point for others who may wish to explore this type of inferencing in the future. Quantitative data storage in the Corvus object model is centered around the *Observation *class. Instances of this class represent individual data points in a collection of data, such as an array. They contain the numerical value of the data as well as pointers to other classes indicating the type and provenance of the data. These other classes include *Dataset*, which holds metadata on experimental conditions; *Measure*, which specifies details about the type of data being measured; *Sample*, which describes the cell line being measured; and *Reporter*, the genomic feature (typically a gene) for which data is being reported. We mapped *Observation *to the IAO class *measurement datum *and used the IAO data property *has measurement value *to associate numerical data values. *Dataset *was linked to the IAO class data set. Individual *Observations *can be specified as belonging to a *Dataset *using the RO property *part of*. Samples were declared as instances of the OBI class *cell culture*. Association of an *Observation *with a *Sample *was done using IAO's is *about *property. *Reporter *was linked to the *Genomic Region *class from the GELO ontology. This class is defined as a superclass of the OBO Sequence Ontology's (SO) [33]*biological region *class and it attaches properties to assign a reference location for a genomic element within the genome. For the purposes of our data, *Reporters *were made instances of SO's *transcript *class, as the reference sequence (RefSeq) was used. We used the URIs for RefSeq sequences provided by the Bio2RDF project. Using this normalized identifier allows us to easily link with other semantic models describing the same genes. Bio2RDF also provides us with an ecosystem whereby a stable link between this and other identifiers is maintained in the absence of a single autoritative global identifier. Similarly, it protects our reference from becoming disconnected from future versions of the same identifier. To capture information from the Corvus *Measure *class, instead of mapping to an instance of a class, we forwarded two of *Measure*'s fields to properties in the domain of *measurement datum*. These were the IAO *is quality measurement of *property and the IAO has *measurement unit label *property. Finally, we used the Dublin Core [34] annotation properties *title *and *identifier *to assign names for *Samples*, *Datasets *and *Reporters *and reference identifiers to *Reporters*. A detailed view of this model is provided in Figure 1.

#### Querying the Corvus SPARQL endpoint

To retrieve a subset of the Corvus warehouse that was sufficient for our ultimate querying purposes, we issued a SPARQL query that would retrieve all relevant information for the seven cell lines mentioned above. We used a SPARQL *DESCRIBE *query which simply returns all relevant properties for a type into a semantic model. Our query retrieves all *Observations *associated with the cell lines and pulls in information on experimental conditions from the *Sample *and *Dataset *tables and on the genes involved from the *Reporter *table. We issued the following SPARQL query for each of the seven cell lines:

PREFIX obo: <http://purl.obolibrary.org/obo/>

PREFIX dc: <http://purl.org/dc/elements/1.1/>

PREFIX ro: <http://www.obofoundry.org/ro/ro.owl#>

DESCRIBE ?rep ?obs ?data ?samp

WHERE {

   ?samp dc:title ''YUMAC''.

   # IAO_0000136 = 'is_about'

   ?obs obo:IAO_0000136 ?samp.

   ?obs ro:part_of ?data.

   ?obs obo:IAO_0000136 ?rep.

}

Retrieval of a populated semantic model containing the approximately 120,000 observation for a cell line using our Hibernate-based mapping typically took between one and two minutes.

### Semantic representations of functional knowledge

#### Annotated GO terms

To include functional information about genes, we decided to incorporate the well-known GO. GO is presented in the OBO format, a simple model for expressing hierarchies of terms and the relationships between them. Although significantly less powerful for inferencing than a fully DL-compatible language like OWL, the OBO language makes it straightforward to declare relationships between classes of object.

We found an effective compromise to be the use of the Simple Knowledge Organization System (SKOS) [35]. In this ontology, written in OWL, terms such as those in OBO taxonomies are expressed as instances of a *Concept *class. Class subsumption is handled though OWL object properties that describe *Concepts *as *broader *or *narrower *than other *Concepts*. In this system, properties can be assigned easily to class-like terms without violating the strictures of OWL-DL. This approach offers significant advantages for querying and reasoning, as the common alternative, creation of restrictions on classes, is computationally expensive while still requiring the creation of individual instantiations to infer properties. Using the OBO to SKOS conversion tools developed at University of Manchester [36], we created a GO-SKOS ontology which converts GO terms to instances of *Concept *and *is *a relationships to *broader *relationships.

**Figure 2:**
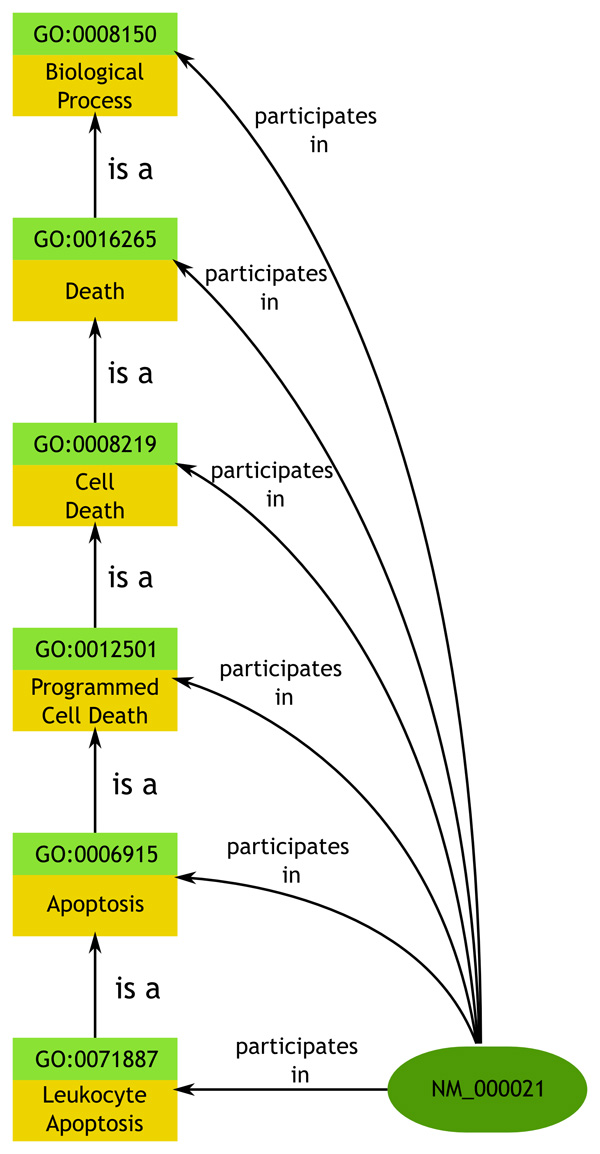
**Gene Ontology annotation hierarchy**. Diagram showing the propagation of the participates_in property up the class subsumption hierarchy. This inference is achieved by using an OWL 2 property chain associating the participates in property with the SKOS broader property.

We downloaded the standard human genome annotations provided by the Gene Ontology consortium. In order to easily merge with the quantitative semantic model retrieved from Corvus, we converted the GO annotation file's HUGO symbols to RefSeq identifiers using conversion tables made available from Entrez [37] and used the Bio2RDF normalized URIs. In fitting with the Corvus model, we cast individual refseqs as instances of the SO:transcript class. We then used three basic relationships from RO to link the gene to its appropriate term in whichever of GO's three main hierarchies. Genes annotated with a Biological Process term were linked using *participates in*; those labeled as expressing a Molecular Function were linked using *has function *and genes marked as being located in a particular Cellular Component were linked using *part of*. We also wished for the properties assigned to genes to propagate up the chain of hierarchy. In other words, if a particular gene participates in a specific biological process, we wanted the reasoner to be able to infer that it also participates in the more generic process. For example, genes participating in apoptosis also participate in the more general process of cell death and in biological processes in general. To accomplish this, we used an OWL property chain, a new feature in OWL 2, to associate *participates in *with *broader*, stating that if A participates in B and C is a broader concept than B, then A participates in C as well. This type of inference is possible because the is-a (subsumption) relationship between SKOS concepts is a relationship between individuals rather than between classes. The relationship is illustrated in Figure 2. We made the same declarations for the *has function *and *part of *properties.

With these declarations in place we were able to run the semantic model through a DL reasoner and create a greatly expanded semantic model with all inferences spelled out (i.e. all annotation properties propagated along the hierarchy). There is a trade-off here as we gain faster query times by precomputing all inferences at the expense of additional storage space and less flexibility, as we need to recompile when the underlying data changes. Creation of the fully entailed GO annotation semantic model took approximately five minutes on our Linux workstation using 8 GB of memory.

#### Transcription factor networks

**Figure 3:**
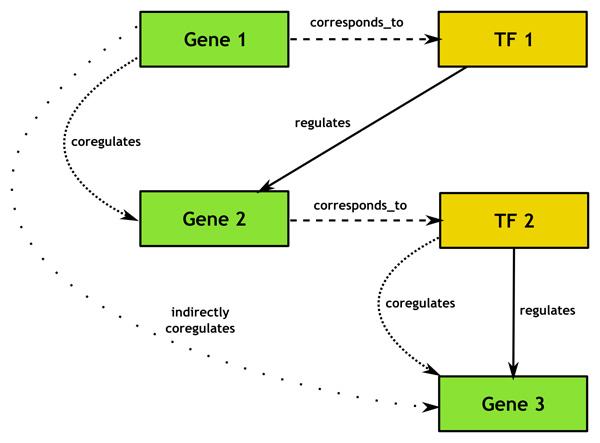
**Transcription factor network properties**. Diagram showing the properties used for describing transcription factor networks. The *corresponds_to *and *regulates *properties are directly stated while the *coregulates *and *indirectly_coregulates *properties are inferred by expansion of OWL 2 property chains.

We retrieved a list of 615 known human transcription factors and the sets of genes they regulate. These sets are commonly recursive as many of the regulated genes are themselves associated with transcription factors. By linking transcription factors to genes and expanding the recursive nodes, we can create a large (and often partially cyclical) network of genes that are within the sphere of influence of a transcription factor. This can be done with relative ease using OWL language constructs and a DL reasoner such as Pellet. We did so by creating a handful of ad-hoc OWL properties and property chains. The custom properties are: *corresponds_to*, which relates a transcription factor to a gene which expresses it; *regulates*, which relates a gene to a transcription factor which regulates its expression; *coregulates*, which relates a gene expressing a transcription factor with the genes that the transcription factor regulates; and *indirectly_coregulates*, which relates a gene expressing a transcription factor to genes further out in the sphere of influence of the transcription factor. A property chain allows us to infer that if transcription factor TF1 *corresponds_to *gene G1 and TF1 *regulates *a second gene, G2, then G1 *coregulates *G2. A second property chain declares that if G1 *coregulates *G2 and G2 *coregulates *a third gene, G3, then G1 *indirectly_coregulates *G3. Running this semantic model through a DL reasoner like Pellet will expand these relationships to closure and result in a network reflecting the full sphere of influence of each transcription factor. Often, these networks will be quite large, ranging into the hundreds and thousands of genes. Creation of the fully entailed transcription factor semantic model took around three minutes on the aforementioned Linux workstation. The relationships used to describe transcription factor networks are illustrated in Figure 3.

### Integrating and using the results

#### Merging of RDF graphs

**Figure 4:**
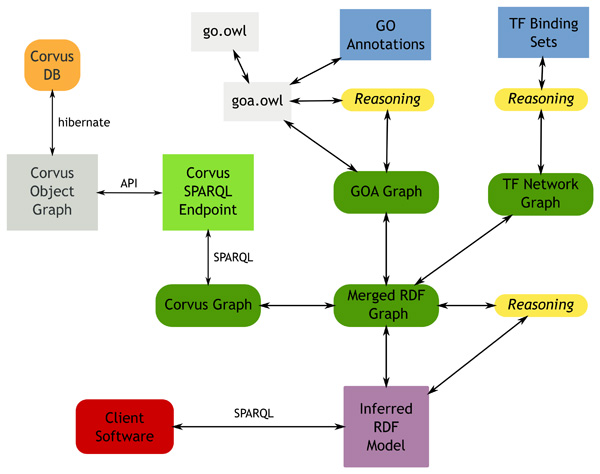
**Model architecture**. Diagram showing the architecture of the integrated model we used to perform the queries in this paper.

The GO annotation and transcription factor network semantic model could at this point be merged with the quantitative semantic model retrieved from Corvus, the points in common being the instances of SO *transcript *representing individual RefSeqs/genes. Because we use identical URIs from the Bio2RDF namespace to describe these instances, we can assure that we are referring to the same gene in the two sources. This merged semantic model could now be queried using SPARQL. The full architecture of our setup for creating an RDF graph from Corvus and merging it with the GO graph is shown in Figure 4.

#### Example queries

We wanted to show that it was possible to use Corvus to execute arbitrarily complex queries incorporating information across varied knowledge domains. To this end, we tried to verify cell lines that were resistant or sensitive to Decitabine, a demethylating agent used for melanoma therapy. Our formulated query asks for genes with high methylation values prior to Decitabine administration and increased gene expression following. We look at the number of such genes involved in apoptosis and compare this with the number involved in an antithetical process - cell proliferation. We use values from two datasets obtained from the Corvus SPARQL endpoint, relative methylation values prior to treatment and ratio of gene expression post-to pre-treatment. Apoptosis- and cell-proliferation-related genes were found by integrating the semantic model containing the GO annotations. Using features from the recently standardized SPARQL 1.1, we can aggregate genes by cell line to get a count of highly expressed genes per cell line. We retrieve the count of methylated and highly expressed genes associated with apoptosis and the ratio of this value of the number of methylated and highly expressed genes associated with cell proliferation. The SPARQL query is:

PREFIX dc: <http://purl.org/dc/elements/1.1/>

PREFIX ro: <http://www.obofoundry.org/ro/ro.owl#>

PREFIX obo: <http://purl.obolibrary.org/obo/>

PREFIX go: <http://purl.org/obo/owl/GO#>

PREFIX k: <http://krauthammerlab.med.yale.edu/>

SELECT (count (distinct ?repA) as ?apopcount) (count (distinct ?repA)/

               count (distinct ?repB) as ?ratio) ?sampName

WHERE {

   ?ds dc:title "Methylation Relative" .

   ?ds2 dc:title "AZA Pre-Post Treatment Ratios" .

   ?obs ro:part_of ?ds .

   # IAO_0000004 = 'has measurement value' ?

   # obs obo:IAO_0000004 ?obsVal .

   ?obs obo:IAO_0000136 ?repA .

   ?obs obo:IAO_0000136 ?samp .

   # OBI_0100060 = 'cell culture'

   ?samp a obo:OBI_0100060 .

   ?samp dc:title ?sampName .

   # go:0006915 = 'apoptosis'

   ?repA ro:participates_in go:0006915 .

   ?obs2 obo:IAO_0000136 ?repA .

   ?obs2 ro:part_of ?ds2 .

   ?obs2 obo:IAO_0000004 ?obsVal2 .

   ?obs2 obo:IAO_0000136 ?samp .

   ?dsB dc:title "Methylation Relative" .

   ?dsB2 dc:title "AZA Pre-Post Treatment Ratios" .

   ?obsB ro:part_of ?dsB .

   ?obsB obo:IAO_0000004 ?obsBVal .

   ?obsB obo:IAO_0000136 ?repB .

   ?obsB obo:IAO_0000136 ?sampB .

   ?sampB a obo:OBI_0100060 .

   ?sampB dc:title ?sampName .

   # go:0008283 = 'cell proliferation'

   ?repB ro:participates_in go:0008283 .

   ?obs2B obo:IAO_0000136 ?repB .

   ?obs2B ro:part_of ?dsB2 .

   ?obs2B obo:IAO_0000004 ?obsBVal2 .

   ?obs2B obo:IAO_0000136 ?sampB .

   FILTER ( ?obsVal > 2) .

   FILTER ( ?obsVal2 > 1) .

   FILTER ( ?obsBVal > 2) .

   FILTER ( ?obsBVal2 > 1) .

}

GROUP BY ?sampName

ORDER BY ?sampName

We can compare these counts to what we know from experimental data regarding the level of sensitivity/resistance of various cell lines [7]. The results are shown in Table 1: The sensitive cell lines with low IC50 values (YUMAC, YUSAC and YULAC) had the three highest gene counts, whereas the two most resistant lines (WW165 and YURIF) had the lowest. Additionally, whereas the resistant cell line WW165 had a low proportion of apoptosis-related genes to cell-proliferation-related genes (0.33), the five sensitive cell lines each show equivalent numbers of apoptosis- and cell-proliferation-related genes. As the mechanism of Decitabine action is demethylation of gene promoters, and (re)expression of the corresponding genes, these results give rise to the following hypothesis: Decitabine targets apoptosis-related gene promoters predominantly in Decitabine-sensitive cell lines, thus conveying its cytotoxic effect by activating the apoptosis pathway. The following validation steps are warranted to strengthen the hypothesis: First, one might want to independently test in vitro both the demethylation of the implicated gene promoters, as well as the re-expression of the corresponding genes. Also, the finding should be repeated in a larger cohort of melanoma samples. A current limitation of our SPARQL query is that we only interrogate for fold change after Decitabine treatment. As shown in prior work, the absolute change in expression values after treatment should also be taken into account [38].

**Table 1:** Apoptosis-related genes by cell line

Cell Line	Apoptosis-Related	Ratio	IC50 (nM)
YUMAC	11	0.916	34
YUSAC2	4	1	91
YULAC	5	1.25	110
YUSIT1	2	2	132
YUGEN8	3	0.6	139
WW165	2	0.333	239
YURIF	0	0	255

Table showing the seven melanoma cell lines, the total number of apoptosis-related genes positively expressed that were formerly methylated, the ratio of these to the number of cell-proliferation-related genes positively expressed that were formerly methylated and the IC50 value.

In attempt to strengthen our hypothesis, we tried to find genes expressing transcription factors which also matched our criteria of being methylated and then highly expressed post-AZA treatment. We issued the following SPARQL query to identify transcription factors and samples for which this was the case:

PREFIX dc: <http://purl.org/dc/elements/1.1/>

PREFIX ro: <http://www.obofoundry.org/ro/ro.owl#>

PREFIX obo: <http://purl.obolibrary.org/obo/>

REFIX k: <http://krauthammerlab.med.yale.edu/>

SELECT distinct ?rep ?tf ?samp ?obsVal ?obsVal2

WHERE {

   ?ds dc:title "Methylation Relative" .

   ?ds2 dc:title "AZA Pre-Post Treatment Ratios" .

   ?obs ro:part_of ?ds .

   ?obs obo:IAO_0000004 ?obsVal .

   ?obs obo:IAO_0000136 ?rep .

   ?obs obo:IAO_0000136 ?samp .

   ?samp a obo:OBI_0100060 .

   ?rep k:corresponds_to ?tf .

   ?obs2 obo:IAO_0000136 ?rep .

   ?obs2 ro:part_of ?ds2 .

   ?obs2 obo:IAO_0000004 ?obsVal2 .

   ?obs2 obo:IAO_0000136 ?samp .

   FILTER (?obsVal > 2) .

   FILTER (?obsVal2 > 0.5)

}

The query identified the transcription factors V$HEN1 01 and V$HEN 02 as formerly methylated and then highly expressed following administration of Decitabine in the YUMAC cell line. As seen above, this is notable as being the cell line most sensitive to Decitabine. The next step was to analyze the degree to which genes regulated by these transcription factors were highly expressed. Table 2 shows the number of genes regulated by each transcription factor for each cell line that are highly expressed. We used the Fisher Exact Test to determine the significance of these results. As can be seen the results for YUMAC are highly significant, much more so than for the other cell lines. Finally, we looked at the proportion of these highly expressed genes regulated by the two transcription factors that are apoptosis-related as opposed to cell-proliferation-related. This is shown in Table 3. As expected, the ratio is higher for YUMAC than for other less sensitive cell lines, suggesting that Decitabine-related promotion of activation of the apoptosis pathway is most pronounced in the cell lines most sensitive to the drug.

**Table 2:** Highly expressed transcription factor regulated genes

Cell line	V$HEN1_01	V$HEN1_02	Total
YUMAC	90 (6.7 × 10^-6^)	108 (9.0 × 10^-16^)	5534
YUSAC2	31 (0.0055)	21 (0.25)	1795
YULAC	19 (0.36)	40 (2.1 × 10^-9^)	1514
YUSIT1	11 (0.022)	8 (0.15)	504
YUGEN8	18 (0.022)	10 (0.73)	977
YURIF	3 (0.64)	6 (0.31)	440
WW165	30 (0.054)	27 (0.091)	4015

Table showing the seven melanoma cell lines; the total number of genes positively expressed that are directly regulated by V$HEN1_01 and V$HEN1_02 along with the P-Value from the Fisher Test comparing this with the overall distribution of highly expressed genes; and the total number of genes positively expressed overall in the cell line.

**Table 3:** Apoptosis to cell proliferation ratios for transcription factor regulated genes

Cell line	V$HEN1_01	V$HEN1_02
YUSIT1	2 (2)	2 (0.67)
YUSAC2	0 (0)	0 (0)
YURIF	3 (1)	3 (1)
YUMAC	10 (0.77)	19 (0.79)
YULAC	3 (0.75)	2 (0.4)
YUGEN8	2 (0.5)	2 (0.5)
WW165	9 (0.64)	7 (2.33)

Table showing the seven melanoma cell lines; the number of positively expressed genes that are associated with apoptosis and the ratio of those genes to the number of positively expressed genes that are associated with cell proliferation for V$HEN1_01 and V$HEN1_02.

## Conclusions

Our proof of concept query illustrates how easily data from various sources can be integrated using the common framework of OWL/RDF. It reveals some of the power of Semantic Web reasoning and querying tools for inferring and elucidating discovered knowledge. It also shows the importance of customization in mapping non-semantic data to RDF. While generic tools mapping relational data to RDF have recently emerged, our experience with d2rq has shown that there are still areas where direct mapping is significantly more efficient and flexible. Our work also makes a strong case for the benefits of using linked data, as use of the Bio2RDF normalized URI for RefSeqs made integration of the two branches of our ontology a breeze.

The flexibility of the Corvus object model will allow us to incorporate quantitative Omics data from a variety of modalities. In the future, this could include cancer data from caArray or caIntegrator or data obtained directly from ArrayExpress using MAGETab2RDF [39]. Essentially, Corvus functions as a contextualized observation repository and we intend to incorporate information from other contexts including clinical data and generic provenance data. We hope to use the new semantic access point to Corvus to integrate this data with other types of information such as pathway and pharmacological data. The simplicity and elegance of the integrated Semantic Web approach also suggests its usefulness as an access point to making sense of variegated data for researchers unequipped with the programming or mathematical expertise to work with traditional data mining tools.

## Methods

### Quantitative data from melanoma cell lines

We examined data derived from seven melanoma cell lines (WW165, YUMAC, YUGEN8, YUSAC2, YUSIT1, YULAC and YURIF). These lines have been experimentally classified using IC50 values from dose-response analysis as being either sensitive to (YUMAC, YUSAC2, YULAC, YUSIT1, YUGEN8) or resistant to (WW165, YURIF) decitabine (5-Aza-2'-deoxy-cytidine, Aza), a DNA methyltransferase inhibitor. Specifically we looked at relative methylation values prior to administration of AZA and the ratio of gene expression following administration of AZA to before. The methylation values were obtained from a Nimblegen promoter array using the Methyl-DNA immunoprecipitation (MeDIP) technique [40,41]. Gene expression ratios were obtained using a custom 2-channel Nimblegen array. Data from both arrays are available for download through ArrayExpress [42]. We used the Gene Element Ontology (GELO) to align the array probes to RefSeq identifiers [43].

### Programming the merged dataset

The Corvus SPARQL endpoint Application Programming Interface (API) was written in Java making extensive use of the Jena API for RDF manipulation and the closely related ARQ API for SPARQL processing [44]. The GO Annotation pre-processing was handled by a Java program making use of the OWLAPI OWL2 library [45] and the Pellet DL reasoner for Semantic Web data [46]. Merging of the semantic models was also handled by Java code using first the ARQ API to issue the SPARQL query on the relational Corvus store and then OWLAPI to perform the actual merge. The merged dataset was loaded into an instance of TDB, an RDF triple store employing the Jena libraries. It was then loaded into a running instance of Joseki, a web application allowing execution of SPARQL queries over HTTP. Joseki also uses the Jena libraries extensively. An endpoint for the merged dataset is available at http://doppio.med.yale.edu:2020/sparql.

## List of abbreviations used

API: Application Programming Interface; BFO: Basic Formal Ontology; caBIG: cancer Biomedical Informatics Grid; DL: Description Logic; ETL: Extraction-Transform-Load; GELO: Gene Element Ontology; GO: Gene Ontology; HTTP: Hyper Text Transfer Protocol; IAO: Information Artifact Ontology; MeDIP: Methyl-DNA immunoprecipitation; OBI: Ontology of Biomedical Investigations; OBO: Open Biomedical Ontologies; ORM: Object-Relational Mapping; OWL-DL: OWL Description Logic; OWL: Web Ontology Language; RDF: Resource Description Framework; RDFS: RDF Schema; RO: Relation Ontology; SKOS: Simple Knowledge Organization System; SO: Sequence Ontology; SPARQL: Simple Protocol and RDF Query Language; SPORE: Specialized Program in Research Excellence; SQL: Structured Query Language; TMA: Tissue Microarray; URI: Uniform Resource Identifier.

## Competing interests

The authors declare that they have no competing interests.

## Authors' contributions

Matthew Holford designed and implemented the SPARQL endpoint to Corvus, the semantic models of functional data and the integrated semantic model used for Decitabine research. He also wrote the paper. Jamie P. McCusker developed the Corvus object model, provided general guidance on development and edited the paper.

Kei-Hoi Cheung provided general design guidance, helped formulate the biomedical queries and edited the paper.

Michael Krauthammer provided research and design guidance, helped formulate the biomedical queries and edited the paper.
